# CFTR promotes malignant glioma development via up‐regulation of Akt/Bcl2‐mediated anti‐apoptosis pathway

**DOI:** 10.1111/jcmm.15300

**Published:** 2020-05-28

**Authors:** Mingyue Zhao, Jieting Zhang, Wenqing Huang, Jianda Dong, Jinghui Guo, Kin Pong U, ZhiHui Weng, Si Liu, Hsiao Chang Chan, Hua Feng, Xiaohua Jiang

**Affiliations:** ^1^ Department of Neurosurgery Airforce General Hospital of the PLA Beijing China; ^2^ Department of Neurosurgery Southwest Hospital Third Military Medical University Chongqing China; ^3^ Epithelial Cell Biology Research Center The Chinese University of Hong Kong Hong Kong SAR China; ^4^ Key Laboratory for Regenerative Medicine of the Ministry of Education of China School of Biomedical Sciences Faculty of Medicine The Chinese University of Hong Kong Hong Kong SAR China; ^5^ Department of Transfusion Medicine Shenzhen Hospital Southern Medical University Shenzhen China; ^6^ Department of Pathology Ningxia Medical University Yinchuan China; ^7^ School of Biomedical Sciences Core Laboratory Shenzhen Research Institute The Chinese University of Hong Kong Shenzhen China

**Keywords:** Akt, apoptosis, Bcl2, CFTR, glioma

## Abstract

Cystic fibrosis transmembrane conductance regulator (CFTR), a cAMP‐activated Cl^‐^ channel, is extensively expressed in the epithelial cells of various tissues and organs. Accumulating evidence indicates that aberrant expression or mutation of *CFTR* is related to carcinoma development. Malignant gliomas are the most common and aggressive intracranial tumours; however, the role of CFTR in the development of malignant gliomas is unclear. Here, we report that CFTR is expressed in malignant glioma cell lines. Suppression of CFTR channel function or knockdown of CFTR suppresses glioma cell viability whereas overexpression of CFTR promotes it. Additionally, overexpression of CFTR suppresses apoptosis and promotes glioma progression in both subcutaneous and orthotopic xenograft models. Cystic fibrosis transmembrane conductance regulator activates Akt/Bcl2 pathway, and suppression of PI3K/Akt pathway abolishes CFTR overexpression–induced up‐regulation of Bcl2 (MK‐2206 and LY294002) and cell viability (MK‐2206). More importantly, the protein expression level of CFTR is significantly increased in glioblastoma patient samples. Altogether, our study has revealed a mechanism by which CFTR promotes glioma progression via up‐regulation of Akt/Bcl2‐mediated anti‐apoptotic pathway, which warrants future studies into the potential of using CFTR as a therapeutic target for glioma treatment.

## 1 INTRODUCTION

Gliomas are the most malignant tumours of central nervous system (CNS) with overall poor outcome and inevitable lethality.^1^ 70%‐80% of gliomas are derived from astrocytes, a kind of star‐shaped glial cells, and therefore are known as astrocytomas. The most malignant Grade IV glioma is defined as glioblastoma multiforme (GBM) and characterized by advanced features of malignancy including vascular proliferation, high metastasis and invasiveness.^2^ Despite decades of enormous efforts in multimodal therapy including surgery, radiation and chemotherapy, the overall 5‐year survival rate of high‐grade malignant glioma patients remains <15%.^3^ This dismal clinical outcome makes gliomas a focus of cancer research.

In the last two decades, molecular genetic studies have identified a number of chromosome or genetic alteration in gliomas, especially in glioblastomas. Several genes, such as *p53*, *p16Ink4a*, retinoblastoma (*RB*), phosphatase and tensin homolog deleted from chromosome 10 (*PTEN*) and epidermal growth factor receptor (*EGFR*), are altered in gliomas.^4^ Among them, genetic alterations of *EGFR* and PI3K/PTEN/Akt pathway appear in 88% of malignant gliomas.^4^, ^5^ Moreover, aberrant activation of PI3K/Akt/mTOR pathway has been correlated with poor prognosis in glioblastoma patients.^6^ The PI3K/Akt/mTOR pathway regulates various cellular functions including survival, metabolism, proliferation and differentiation via a number of downstream effectors such as CREB, p27, FOXO, p70 and 4EBP1.^6^ On the other hand, the pathway is antagonized by various factors including PTEN and GSK3β to prevent it from over‐activation, which subsequently leads to dysregulated cellular behaviours, such as apoptosis evasion and uncontrolled cell growth. Indeed, the PI3K/Akt/mTOR pathway is over‐activated in various cancers; therefore, the pathway is an attractive therapeutic target because it functions as a convergence point for divergent growth stimuli and regulates cellular processes that are involved in the initiation and maintenance of cancer.

Cystic fibrosis transmembrane conductance regulator (CFTR) is a cAMP‐activated chloride channel, mutations of which lead to the most common lethal genetic disease.^7^ The correlation between CFTR dysfunction and incidence of cancer has been reported for long time. Large cohort studies have reported an increased risk of overall cancer predisposition in CF patients in North America and Europe.^8^, ^9^ In addition, reduced expression level of CFTR has been observed in various types of cancer including lung cancer, colon cancer and breast cancer..^10^, ^11^, ^12^, ^13^, ^14^ Indeed, various studies have revealed that in a variety of carcinomas, CFTR functions as a tumour suppressor, loss of which promotes the malignant features of cancer cells and cancer development.^10^, ^11^, ^12^, ^13^ However, up‐regulation of CFTR has also been reported, at which CFTR promotes cancer development in female reproduction system.^15^, ^16^ Thus, while CFTR has been implicated in the pathogenesis of cancer development, the exact role of CFTR in cancer is still controversial.

Cystic fibrosis transmembrane conductance regulator was originally found to be expressed in numerous epithelial tissues, such as lung, pancreas, gastrointestinal tract and reproductive tract^17^; however, CFTR expresses in other cell types and tissues as well.^18^ In particular, both RT‐PCR and immunohistochemistry assays demonstrated the prevalent and abundant expression of CFTR in the neurons, but not astrocytes in human brain.^19^ Similarly, *CFTR* mRNA was identified in astrocytes isolated from rat brain.^20^ While the physiological role of CFTR in the brain is unclear, it is suggested that CFTR might be critical for the regulation of chloride homeostasis in the CNS.^21^ In addition, loss of CFTR causes dysfunction of schwann cells and changes in peripheral nervous system (PNS) similar to those phenotypes manifested in Charcot‐Marie‐Tooth disease in *CFTR*‐deficient pigs at birth.^22^ While CFTR has been implicated in cancer development, the role of CFTR in non‐epithelium cell‐derived tumours, like malignant gliomas, is completely unknown. Therefore, we undertook the present study to determine the role of CFTR in malignant gliomas using cell lines, xenograft mouse models and human samples. Our results clearly indicate that CFTR promotes glioma development via Akt/Bcl2‐mediated anti‐apoptosis pathway.

## 2 MATERIALS AND METHODS

### 2.1 Cell lines and treatment

Human glioblastoma cell lines U87‐MG (ATCC, HTB‐14) and U373 (ATCC, HTB‐17) were cultured in DMEM medium whereas U138 (ATCC, HTB‐16) was cultured in EMEM medium (GIBCO, NY, USA). Human glioma cell lines (Grade III) SW1088 (ATCC, HTB‐12) and SW1783 (ATCC, HTB‐13) were cultured in L15 medium (GIBCO, NY, USA). Media were supplemented with 10% foetal bovine serum (FBS). Glioblastoma cell lines were maintained in 5% CO_2_ at 37°C. SW1088 and SW1783 were maintained in air at 37°C. Cells were seeded in 6‐well plates with 5 × 10^4^/well and supplemented with 1% FBS. CFTRinh‐172 (Sigma, MO, USA) or Akt inhibitor, and MK‐2206 2HCL (Sellecchem, TX, USA) was used at the final concentration of 10 µmol/L. PI3K specific inhibitor, LY294002 (Sigma, MO, USA), was used at the final concentration of 10 µmol/L. DMSO (0.1%) was used as vehicle control.

### 2.2 CFTR knockdown and overexpression

For CFTR knockdown, we used miRNA duplex which was synthesized by Life Technologies (Carlsbad, CA). The miRNA sequence is 5′‐TTG GAA AGG AGA CTA ACA AGT‐3′, whereas miRNA against LacZ is used as the control. SW1088 cells were seeded in 35 mm culture dishes, and 3 μg vectors were mixed with 6 μL Lipofectamine 2000 (Invitrogen) following the manufacturer's instruction. Cell lysates were collected 48 hours after transfection for Western blot to confirm the transfection efficiency. The stable transfectants were maintained in medium containing 4 μg/mL blasticidin S.

Wild‐type CFTR and GFP control vectors were kindly provided by professor Tzyh‐Chang Hwang (University of Missouri—Columbia). One day before transfection, U87 cells were seeded at 70%‐80% confluence, and 3 μg plasmid was mixed with 6 μL Lipofectamine 2000 for transfection according to the manufacturer's instruction. After 48 hours of incubation, cells were selected by G418 at the concentration of 1200 μg/mL. The positive selections were maintained in 600 μg/mL G418 afterward.

### 2.3 Whole‐cell patch‐clamp recording

SW1088 cells were cultured on coverslips before patch‐clamp recording. Borosilicate glass‐made patch pipettes (Vitrex, Modulohm A/S, Herlev, Denmark) were filled with pipette solution and pulled with micropipette puller to a resistance of 5‐7 MΩ. Ionic current was recorded with a data acquisition system (DigiData 1322A; Axon Instruments) and an amplifier (Axopatch‐200B; Axon Instruments, Foster City, CA, USA). To evaluate CFTR function, cells were bathed in solution: NaCl 130 mmol/L, KCl 5 mmol/L, MgCl_2_ 1 mmol/L, CaCl_2_ 2.5 mmol/L and Hepes 20 mmol/L with D‐mannitol compensated for OSM 310 (pH 7.4); pipettes were filled with solution: CsCl 101 mmol/L, EGTA 10 mmol/L, HEPES 10mM, TEACl 20 mmol/L, MgATP 2 mmol/L, MgCl_2_ 2 mmol/L, glucose 5.8 mmol/L, and PKA subunit 100 U/mL with D‐mannitol compensated for OSM 290 or NMDG‐Cl 140 mmol/L, HEPES 20 mmol/L, EGTA 10 mmol/L and MgSO_4_ 1 mmol/L with D‐mannitol compensated for OSM 290. The whole‐cell current was obtained by voltage clamp with the commanding voltage elevated from −100 mV to +100 mV with 20 mV increment.

### 2.4 Analyses of cell viability, cell cycle and apoptosis

Cell viability was analysed by MTS assay. 3000‐4000 cells/well were seeded into 96‐well plates. Following the indicated incubation time, media was removed and 100 μL full media with 20 μL MTS (3‐(4,5‐dimethylthiazol‐2‐yl)‐5‐(3carboxymethoxyohenyl)‐2‐(4‐sulfophenyl)‐2H‐tetrazolium, Promega) was added. The experimental plates were incubated for another 2 hours. Then, the intensity of soluble product was measured by spectrophotometer at 490 nm. At least triplicate wells were used for each condition. Y‐axis is calculated using the following equation: day 1‐7 absorbance/day 0 absorbance and presented as folds relative to day 0. Cell cycle and apoptosis were evaluated by flow cytometry on BD FACSAria. For cell cycle analysis, the cells were fixed and stained by propidium iodide. The eBioscience™ Annexin V Apoptosis Detection Kit (Thermo Fisher Scientific) or TUNEL Detection Kit (Thermo Fisher, Scientific) were used for apoptosis assay following the standard protocol.

### 2.5 Reverse transcription‐polymerase chain reaction (RT‐PCR) analysis

Total RNA was isolated by TRIZOL, and 2 μg RNA was used to synthesize cDNA following the manufacturer's protocol (Promega, Madison, WI, USA). Real‐time RT‐PCR reactions were performed using the SYBR Green PCR kit (Takara, Kusatsu) and a 7500 Fast Real‐Time PCR System (Applied Biosystems). The primers were listed in Table S1. The relative mRNA expression of interested genes was indicated with 2^(−ΔΔCt)^.

### 2.6 Western blot analysis

Cell or tissue protein were extracted using lysis buffer (RIPA: 50 mmol/L Tris, 150 mmol/L NaCl, 1% Triton X‐100, 0.1% SDS and 1% sodium deoxycholate, pH 7.4) with protease inhibitors phenylmethylsulphonyl fluoride and pimix. The protein samples were incubated with RIPA and SDS buffer for CFTR detection at room temperature for 30 minutes, or boiled for 3 minutes at 100°C for other protein. Total lysates of cells (20 µg per lane, 60 µg per lane for CFTR detection) or tissues (40 µg per lane) were subjected to SDS‐polyacrylamide gel electrophoresis and transferred onto nitrocellulose. The transferred membranes were blocked with 5% non‐fat dry milk in TBS containing 0.2% Tween‐20 (TBST). Then, the membranes were incubated with primary antibodies as follow: anti‐CFTR (Alomone Labs ACL‐006; or Millipore M3A7; US), anti‐mTOR (Millipore 04‐385; US), anti‐PI3K p110a (Cell Signaling Technology 4249; US), anti‐Akt (Cell Signaling Technology 9272; US), anti‐p‐Akt(ser473) (Cell Signaling Technology 9271; US), anti‐p53 (Cell Signaling Technology 9282; US), anti‐cleaved caspase‐3 (Cell Signaling Technology 9661; US) and anti‐Bcl2 (Santa Cruz 492; US) at 4°C overnight. The membranes were washed with TBST and subsequently incubated with peroxidase‐conjugated secondary antibodies for 1 hour. Eventually, the membranes were washed with TBST and detected by enhanced ECL (Amersham, Piscataway, NJ, USA).

### 2.7 Immunofluorescent staining and immunohistochemistry staining

Glioma cells were seeded into 24‐well plates with cover slips for 24 hours. Cells were fixed with 4% paraformaldehyde. After that, samples were blocked with 1% bovine serum albumin and incubated with primary antibody against CFTR (Alomone Labs, ACL‐006, US) at 4°C overnight with a dilution of 1:100 in 1% BSA. After that, cells were incubated with Alexa fluor 568 antimouse IgG and/or fluor 488 anti‐rabbit IgG at a dilution of 1:500 for 1 hour. Nuclei were co‐stained with Hoechst and the slides were mounted with ProLong Gold Antifade Reagent (Invitrogen) and visualized with fluorescent microscope (Nikon Intersilight C‐HGF1).

IHC analysis of CFTR was conducted according to a previously described method.^23^ Briefly, the paraffin‐embedded tissue sections were baked at 65°C for 2hours, dewaxed with xylenes and then rehydrated with graded ethanol to distilled water. After that, the sections were boiled in EDTA antigen retrieval buffer (pH 8.0) for antigen retrieval. Subsequently, the slides were blocked with 0.3% H_2_O_2_ and normal goat serum, and incubated with CFTR antibody (1:100; Alomone Labs; ACL‐006) at 4°C overnight. After washing with PBST, the tissue sections were incubated with a biotinylated anti‐rabbit secondary antibody. Eventually, the slides were incubated with streptavidin horseradish peroxidase complex at 37°C for 30 minutes and developed with diaminobenzidine tetrahydrochloride (DAB). The tissue sections were evaluated and scored by the intensity of the staining. The staining intensity scores were determined as ‐ (no staining), + (weak staining exhibited as light yellow), ++ (moderate staining exhibited as yellow‐brown) or +++ (strong staining exhibited as brown). The IHC results were analysed by two independent pathologists.

### 2.8 Animal models

Six‐ to eight‐week‐old male nude mice were provided by the Laboratory Animal Service Center of the Chinese University of Hong Kong. They were maintained in an air‐conditioned room with controlled temperature of 24 ± 2°C and humidity of 55 ± 15%, in a 12 hour light/darkness cycle regulation and were fed laboratory chow and water ad libitum. All animal experiments were conducted in accordance with the University Laboratory Animals Service Center's guidelines with approval from the Animal Ethnics Committee of the University. The mice were randomly divided into four groups with 6 mice in each group. Mice were subcutaneously injected with 0.75 × 10^6^ or intracranially injected with 0.2 × 10^6^ control or CFTR‐overexpressing U87 cells separately. The tumour size was calculated according to the following formula: 0.5234 × (long diameter (short diameter)^2^). Two to three weeks after injection, all animals were sacrificed. Tumour weight and size were measured along with the experiments.

### 2.9 Statistical analysis

Statistical significance between two measurements was determined by unpaired Two‐tailed Student's *t* test. One‐way ANOVA and Tukey's post hoc test were used when there were more than two groups. All statistical analyses were conducted by Prism 5 (GraphPad Inc, San Diego, CA, USA). Values of *P* < .05 were considered as statistically significant. All statistical data were shown as mean ± SD.

## 3 RESULTS

### 3.1 Expression and channel function of CFTR in glioma cell lines

**FIGURE 1:**
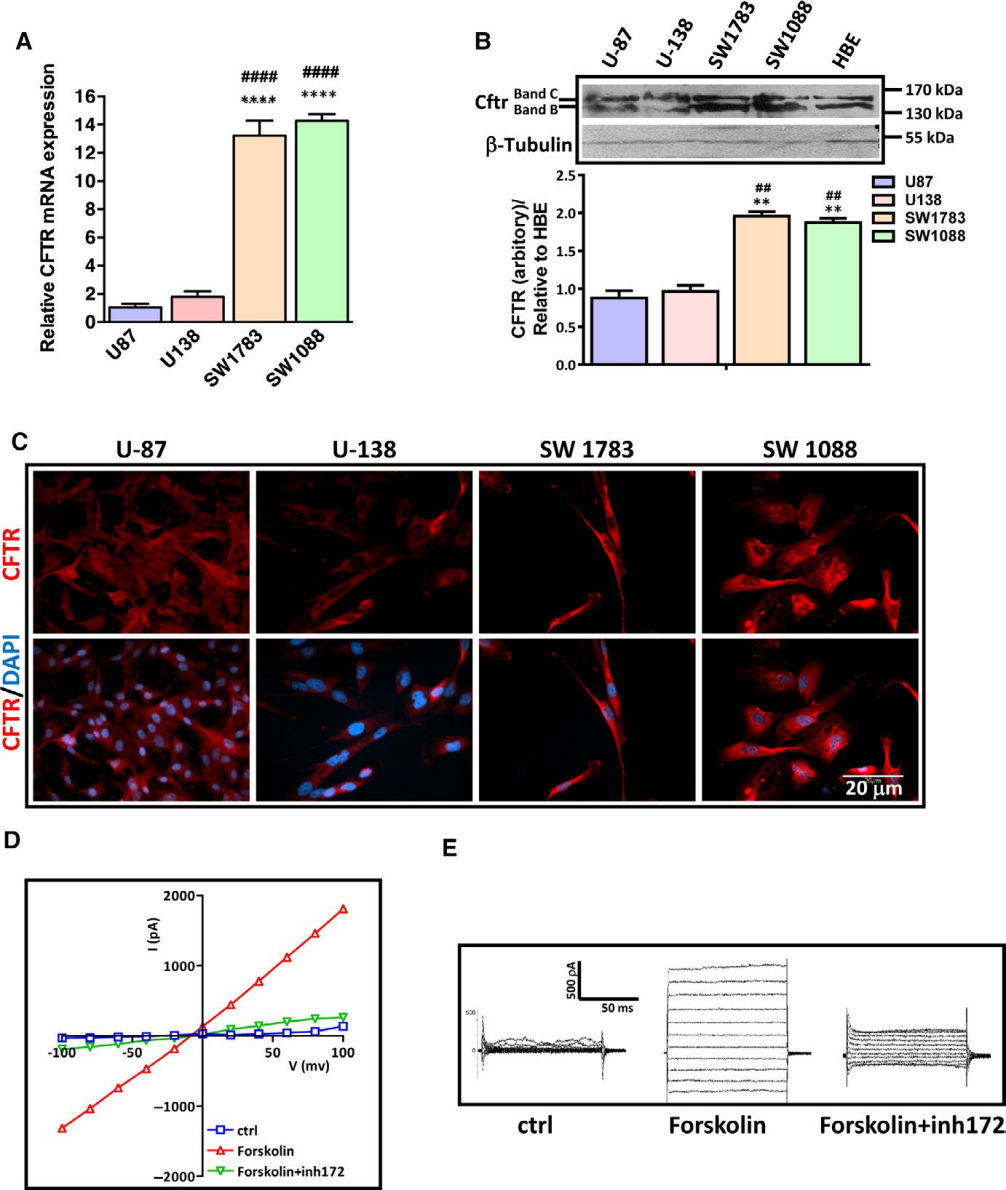
Expression and channel function of cystic fibrosis transmembrane conductance regulator (CFTR) in glioma cell lines. A, Real‐time qPCR of *CFTR* in different glioma cell lines. ****P < .0001 vs U87, ^####^ *P* < .0001 vs U138. Data are mean ± SD, n = 3. B, Representative Western blot shows that the expression of CFTR in glioma cell lines. Note that Western blot result showing both mature, fully glycosylated form of CFTR (Band C) and immature, core‐glycosylated CFTR (Band B). Anti‐C‐terminal‐CFTR was used (Alomone Labs). Human bronchial epithelial, which is a human bronchial epithelial cell line that has been extensively used for cystic fibrosis research, was used as a positive control in Western blot. Quantification of total CFTR is shown as mean ± SD, n = 3. **P < .01 vs U87, ^##^ *P* < .01 vs U138. C, Immunofluorescent staining shows that CFTR (in red) is expressed in glioma cell lines. Cystic fibrosis transmembrane conductance regulator is mainly located in the cytoplasm of glioma cells, scale bar = 20 μm. D, A time‐ and voltage‐independent whole‐cell current in response to forskolin (10 μmol/L) was detected in SW1088 cells. The current could be repressed by inhCFTR‐172 (10 μmol/L). E, Linear I‐V relationship characteristic of CFTR in SW1088. Data are shown as mean ± SD, n = 3

We first examined the expression levels of CFTR in different glioma cell lines (GBM cell lines U138 and U87, and grade III glioma cell lines SW1088 and SW1783) by real‐time PCR. The result demonstrated that *CFTR* was expressed in all malignant glioma cell lines, whereas the expression levels of *CFTR* were higher in SW1783 and SW1088 than that in U87 and U138 (Figure 1A). Two different CFTR antibodies targeting either C terminus (CFTR‐C) or N‐terminus (CFTR‐N) were used to detect CFTR protein in glioma cell lines. Our Western blot result showed that both band B and band C, which indicate the immature and mature isoforms of CFTR, respectively, could be detected by CFTR‐C in glioma cell lines. While the expression levels of mature CFTR were similar in all glioma cell lines, the expression levels of immature and total CFTR were significantly higher in SW1783 and SW1088 (Figure 1B). Consistently, the expression of mature CFTR did not show significant difference among the glioma cell lines with CFTR‐N antibody, which only detects the mature band (170kD) (Figure S1A). The immunofluorescent staining result showed that CFTR was mostly expressed in the cytoplasm of glioma cells (Figure 1C). To determine whether CFTR has channel function in glioma cells, we used patch‐clamp technique to examine CFTR whole‐cell current in SW1088 cells. As shown in Figure 1D, A whole‐cell current in response to an adenylyl cyclase activator forskolin (10 μmol/L) was detected in SW1088 cells, which was time‐ and voltage‐independent. The current could be repressed by CFTR_inh_‐172 (10 μmol/L), a specific CFTR inhibitor (Figure 1D), and showed the linear I‐V relationship characteristic of CFTR^24^ (Figure 1E). These findings indicate CFTR is expressed and functional in malignant glioma cells.

### 3.2 CFTR suppresses apoptosis in glioma cells

**FIGURE 2:**
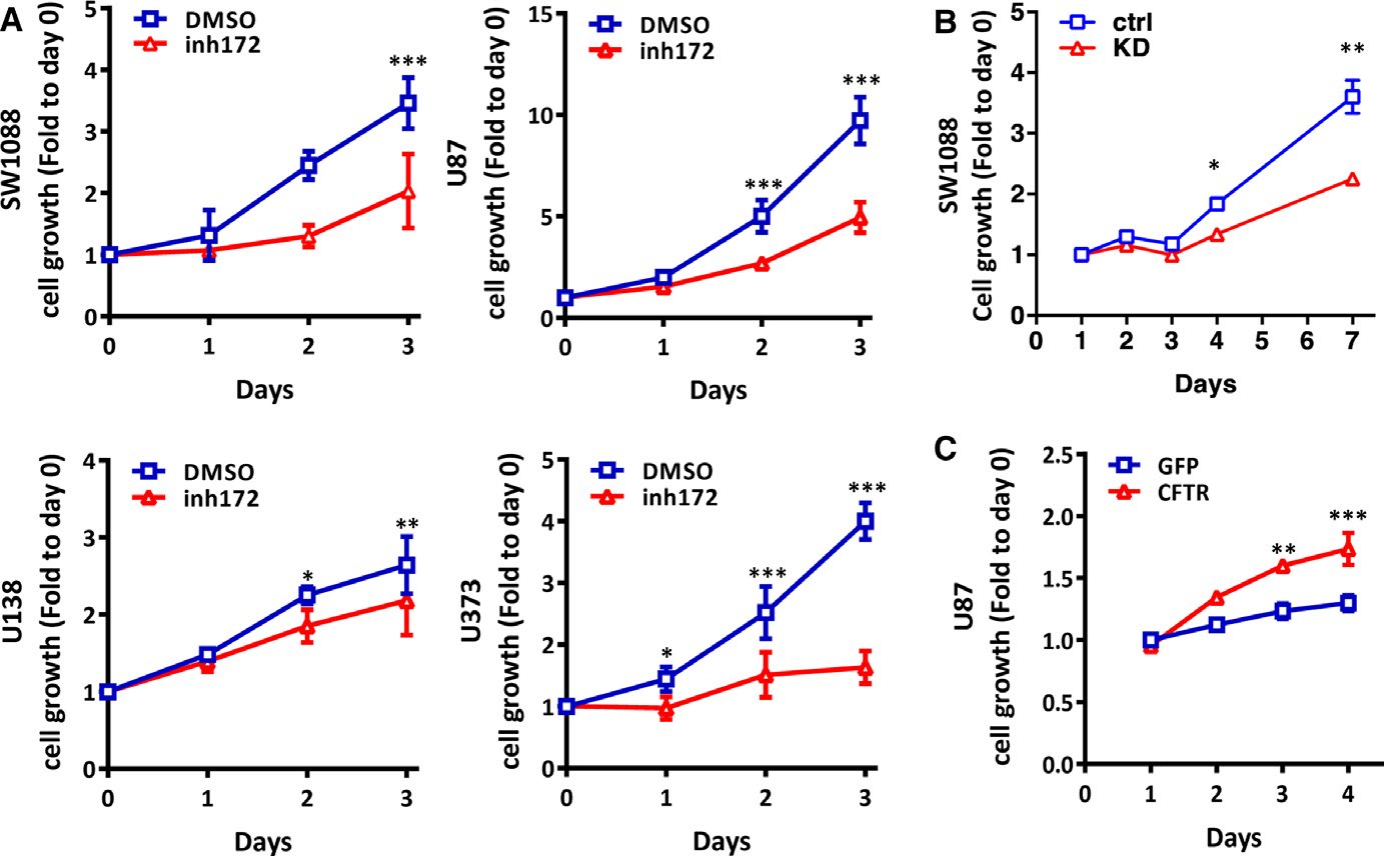
Cystic fibrosis transmembrane conductance regulator (CFTR) affects cell growth in glioma cell lines. A, MTS results show that when treated with 10 μmol/L CFTR specific inhibitor, CFTRinh‐172 (inh172), glioma cell viability is repressed in SW1088, U87, U138 and U373. Data are mean ± SD, n = 3, **P < .01, ***P < .001. B, MTS result shows that knockdown of *CFTR* suppresses cell growth in SW1088 cells, comparing to control cells. Data are mean ± SD, n = 3, *P < .05, **P < .01. C, MTS result shows that overexpression of CFTR enhances cell viability in U87. Data are mean ± SD, n = 3, **P < .01, ***P < .001

We went further to examine whether dysfunction of CFTR affects glioma cell viability. We treated glioma cells with CFTR_inh_‐172 (inh172) and found that CFTR inhibitor repressed cell viability in all glioma cell lines tested (Figure 2A). To further determine the involvement of CFTR in glioma cell viability, we did gain and loss of function studies in U87 and SW1088 cells (Figure S1B,C). Our results showed that knockdown of CFTR significantly reduced cell viability in SW1088 (Figure 2B), while overexpression of CFTR significantly enhanced cell viability in U87 (Figure 2C), indicating CFTR promotes glioma cell viability. We reasoned that the enhanced cell viability could be due to increased cell proliferation or decreased apoptosis. We first determined whether overexpression of CFTR promoted cell proliferation in U87 cells. The result showed that there was no significant difference in PCNA expression between CFTR‐overexpressing and control U87 cells (Figure S1D). In addition, cell cycle analysis demonstrated that neither overexpression nor inhibition of CFTR regulated cell cycle progression in U87 cells (Figure S1E,F). These results indicate that CFTR does not affect cell proliferation in glioma cells.

**FIGURE 3:**
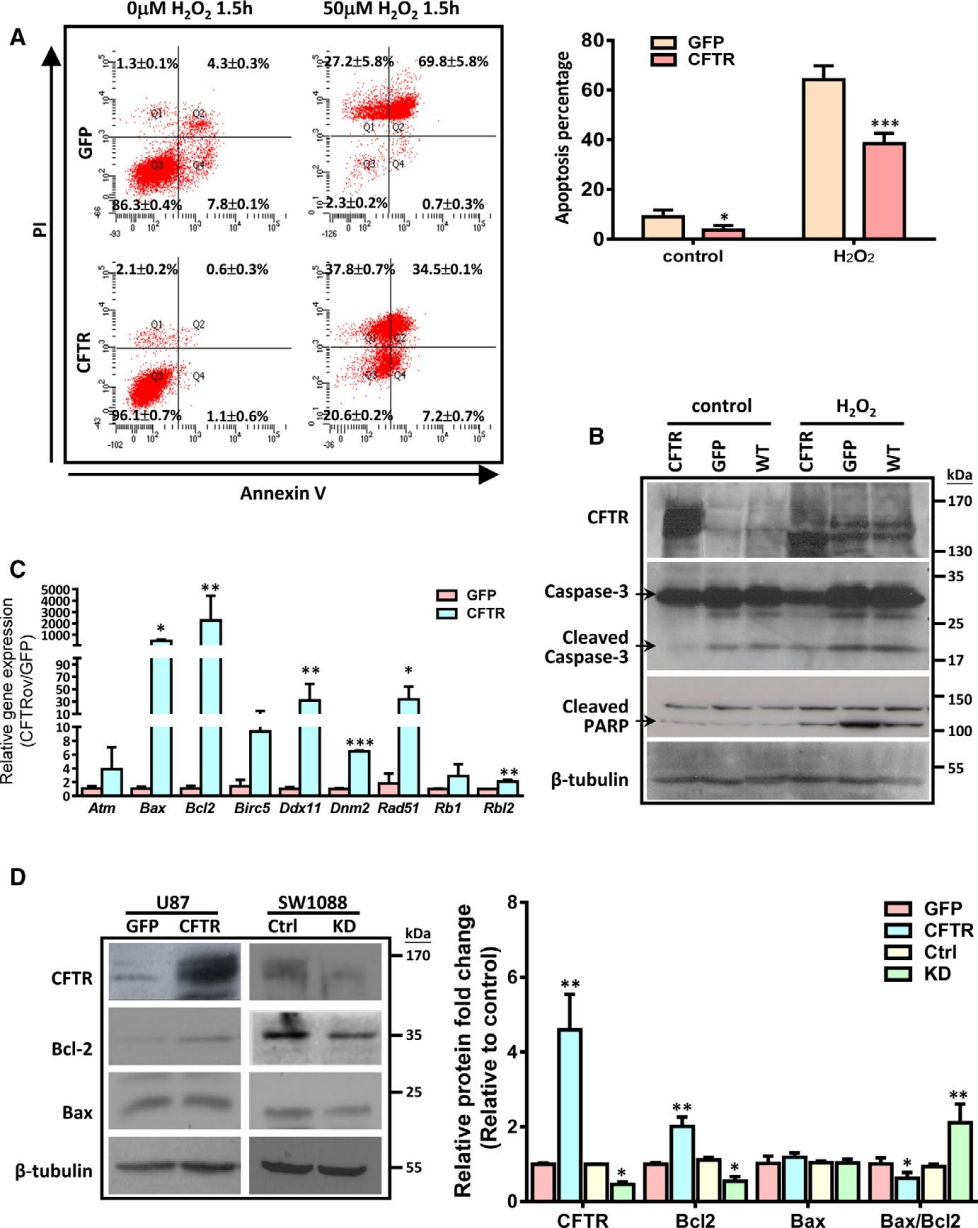
Cystic fibrosis transmembrane conductance regulator (CFTR) regulates apoptotic response in glioma cell lines. A, Flow cytometry data show that overexpression of CFTR suppresses apoptosis on basal level and when challenged by H_2_O_2_ (50 µmol/L) in U87. Quantification analysis of Q2 plus Q4 which indicate both early and late apoptotic cells is shown as mean ± SD from three independent experiments, *P < .05, ***P < .001. B, Representative Western blot result shows that CFTR overexpression suppresses the cleavage of caspase‐3 and PARP in U87 cells treated with H_2_O_2_. The experiments have been repeated three times independently. C, Focused PCR array comparing control and CFTR‐overexpressing U87 cells shows that apoptotic related genes are up‐regulated. Quantification analysis of data is expressed as the mean ± SD from three independent experiments, *P < .05, **P < .01. D, Representative Western blot result shows that overexpression of CFTR enhances the expression of Bcl2 in U87 cells, while knockdown of CFTR reduces the expression of Bcl2 in SW1088 cells. Quantification data is shown on the right, *P < .05, **P < .01

Next, we evaluated whether CFTR had any effect on cell apoptosis. Our flow cytometry result showed that overexpression of CFTR suppressed apoptosis at the basal level (Figure 3A). Moreover, overexpression of CFTR significantly alleviated H_2_O_2_‐induced apoptosis in U87 cells. In particular, while more than 60% cells were Annexin V^+^ in control U87 cells in response to H_2_O_2_, only about 40% of CFTR‐overexpressing U87 cells were apoptotic (Figure 3A). In line with this result, the expression levels of cleaved caspase‐3 and cleaved‐PARP were down‐regulated in CFTR‐overexpressing U87 cells compared to their counterparts upon H_2_O_2_ treatment (Figure 3B). These results indicate that CFTR‐overexpressing glioma cells exhibit a survival advantage. Further characterization using control‐ and knockdown‐SW1088 cells indicated that knockdown of CFTR significantly aggravated apoptotic response to H_2_O_2_ (Figure S2). As a first step to identify the genes that might contribute to the enhanced survival of CFTR‐overexpressing U87, we used a focused real‐time PCR array(PAHS‐020C) including 84 genes that are associated with cell proliferation and apoptosis. As shown in Figure 3C, apoptosis‐related genes, including *Atm, Bax, Bcl2, Birc5, Ddx11, Dnm2, Rad51, Rb1 and Rbl2* were up‐regulated in CFTR‐overexpressing U87 cells, and the most up‐regulated gene is *Bcl2*. To confirm this result, we determined the protein expression of Bcl2 in U87 and SW1088 with CFTR manipulation. Our results showed that overexpression of CFTR enhanced the expression of Bcl2 in U87 cells, while knockdown of CFTR reduced the expression of Bcl2 in SW1088 cells (Figure 3D). In addition, the ratio of Bax/Bcl2 which indicates cell susceptibility to apoptosis was significantly decreased in CFTR‐overexpressing cells whereas increased in CFTR‐knockdown cells. These results indicate that CFTR regulates the responsiveness to apoptotic signal via Bcl2‐mediated anti‐apoptosis pathway in glioma cells.

### 3.3 CFTR promotes glioma progression and suppresses apoptosis in vivo

**FIGURE 4:**
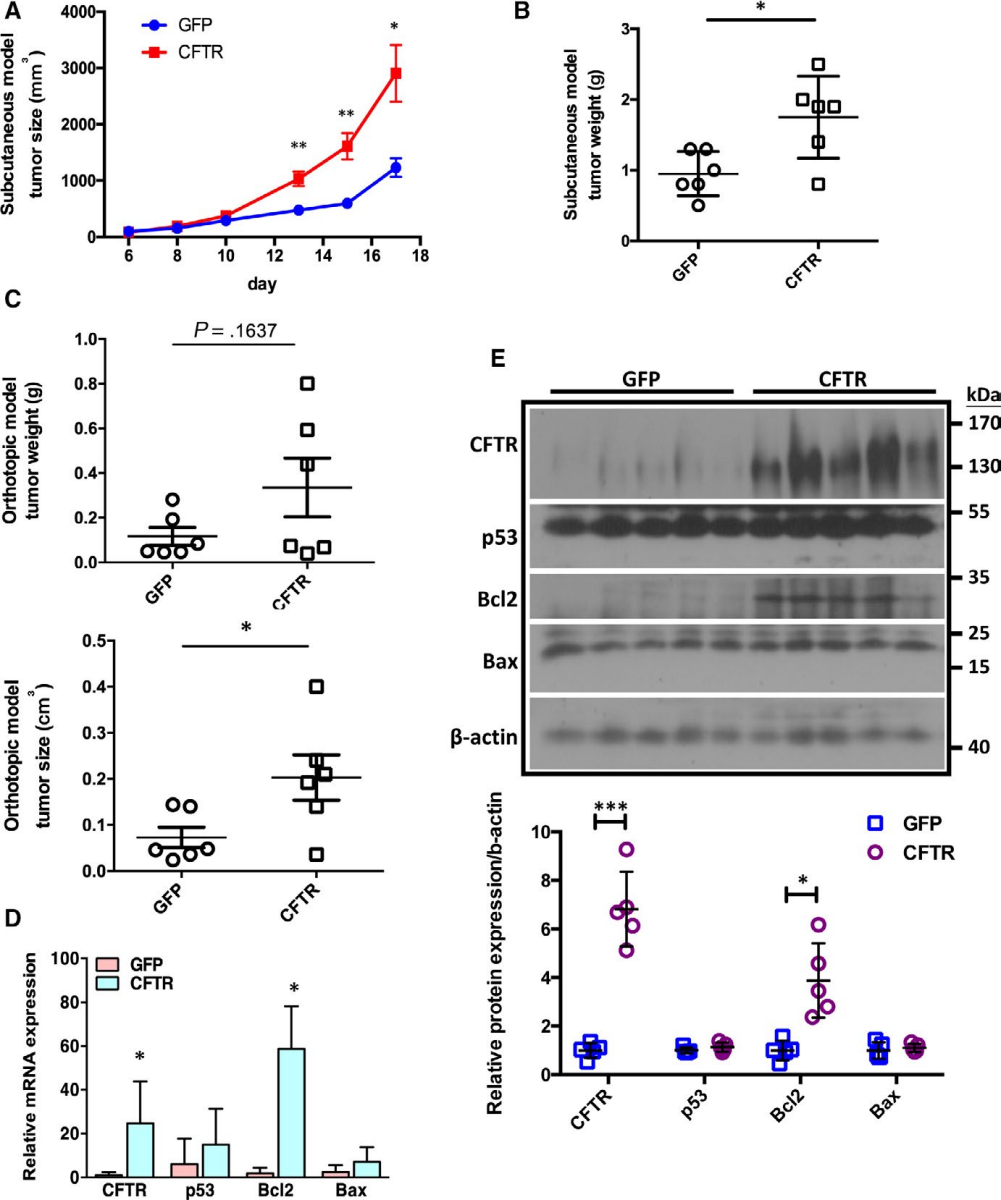
Overexpression of Cystic fibrosis transmembrane conductance regulator (CFTR) promotes tumour formation in vivo. A, Tumour growth kinetics in CFTR‐overexpressing and control U87 cells. n = 6 mice in each group, *P < .05, **P < .01. B, Tumour weight is significantly higher in the tumours formed by CFTR‐overexpressing cells compared to the tumours formed by control cells. n = 6 mice in each group, *P < .05. C, Similar results are shown in orthotopic tumours transplanted with CFTR‐overexpressing or control U87 cells. After 3 weeks, the tumours formed by CFTR‐overexpressing U87 cells show heavier tumour weight (left) and significantly larger tumour size (right), when comparing to the tumours formed by control cells. n = 6 mice in every group, * *P* < .05. D, Real‐time polymerase chain reaction showing the mRNA expression levels of *p53*, *Bcl2* and *Bax* in xenografts formed by CFTR‐overexpressing and control U87 cells. n = 6 mice in each group, *P < .05. E, Western blot assay showing that the expression levels of Bcl2 are up‐regulated in CFTR‐overexpressing tumours compared to the control ones. Quantitation of relative protein expression level is shown below, *P < .05, ***P < .001

To test whether CFTR has similar function in vivo, we established both subcutaneous and orthotopic xenograft models using control or CFTR‐overexpressing U87 cells to evaluate the role of CFTR in glioma progression. In the subcutaneous model, the tumour size was about 3 times and the tumour weight was more than 2 times in CFTR‐overexpressing tumours than control tumours (Figure 4A,B; Figure S3A). Consistently, in orthotopic model, the tumour size was significantly increased in CFTR‐overexpressing tumours compared to control cell‐inoculated tumours as well (Figure 4C; Figure S3B). Subsequent detailed examination of tumour samples revealed that the mRNA expression level of *Bcl2* was significantly up‐regulated in CFTR‐overexpressing tumours (Figure 4D). Our Western blot results also showed that the expression of Bcl2 was significantly up‐regulated in CFTR‐overexpressing U87‐inoculated tumours compared to the tumours injected with control U87 cells (Figure 4E). These results suggest that similar to the findings in vitro, overexpression of CFTR promotes glioma progression by up‐regulation of Bcl2‐mediated anti‐apoptosis pathway in vivo.

### 3.4 CFTR suppresses apoptosis by up‐regulation of Akt/Bcl2 pathway

**FIGURE 5:**
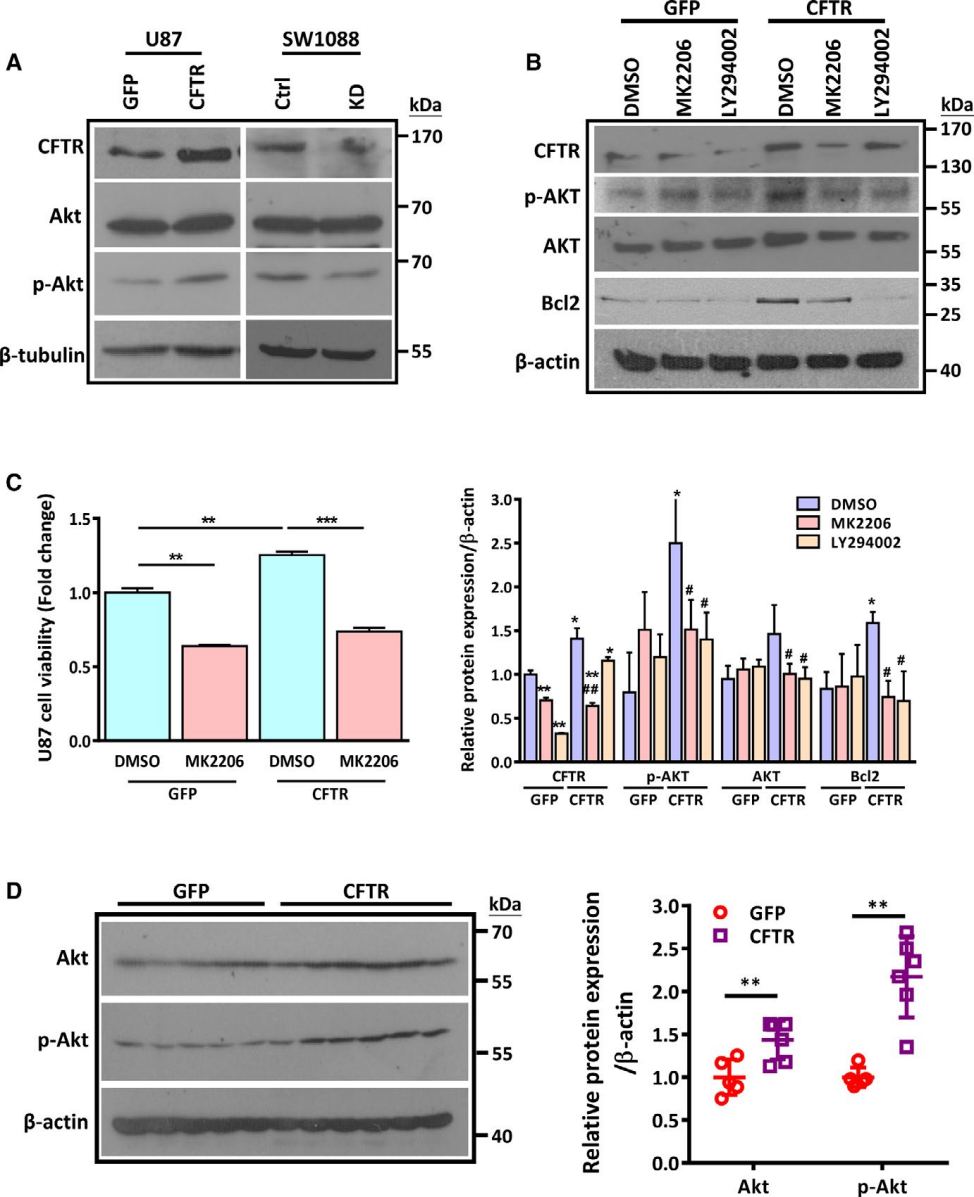
Akt/Bcl2 mediates the role of Cystic fibrosis transmembrane conductance regulator (CFTR) in apoptosis regulation. A, Representative Western blot result shows that overexpression of CFTR increases the phosphorylation of Akt (Ser473) in U87cells, while knockdown of CFTR decreases the phosphorylation of Akt (Ser473) in SW1088 cells. The experiments have been repeated three times independently. B, Western blot analysis shows that either MK2206 (10 µmol/L) or LY294002 (10 µmol/L) reverses CFTR overexpression–induced increase of Bcl2 and p‐Akt. Quantification analysis of data is expressed as the mean ± SD from three independent experiments. Relative to GFP‐DMSO group: *P < .05, **P < .01, ***P < .001; relative to CFTR‐DMSO group: ^#^ *P* < .05, ^##^ *P* < .01. C, MTS result shows that MK2206 reverses CFTR overexpression–induced enhancement of cell viability in U87 cells. Quantification analysis of data is expressed as the mean ± SD from three independent experiments, **P < .01, ***P < .001. D, Western blot assay shows that the accumulation of p‐Akt in xenografts formed by CFTR‐overexpressing U87 cells comparing to xenografts formed by control cells. Quantitation of relative protein expression level is shown in the right panel. n = 6 mice in each group, **P < .01

How does CFTR regulate Bcl2‐mediated anti‐apoptosis pathway? As Akt is known as an upstream regulator of p53/Bax and Bcl2‐mediated apoptotic pathway,^25^ and PI3K/PTEN/Akt pathway appears as most frequently altered signalling pathway in malignant gliomas,^2^, ^26^ we speculated that CFTR might suppress apoptosis via PI3K/Akt pathway. Indeed, our Western blot results showed that overexpression of CFTR enhanced the phosphorylation of Akt (p‐Akt) at Ser473 in U87 cells (Figure 5A, left panel). On the contrary, knockdown of CFTR suppressed the expression level of p‐Akt in SW1088 (Figure 5A, right panel). If the role of CFTR in anti‐apoptosis is mediated by activation of PI3K/Akt pathway, inhibition of PI3K/Akt signalling should abolish the effect of CFTR. Interestingly, either suppression of Akt pathway by a specific p‐Akt inhibitor, MK‐2206 or suppression of PI3K activity by a PI3K specific inhibitor, LY294002 alleviated the up‐regulation of Bcl2 by overexpression of CFTR in U87 cells, indicating CFTR regulates Bcl2 expression via PI3K/Akt pathway (Figure 5B). Moreover, suppression of Akt activity by MK‐2206 rescued the promoting effect of CFTR on cell growth in U87 (Figure 5C). Apart from that, the regulatory effect of CFTR on PI3K/Akt pathway is validated in our xenograft model, which showed up‐regulation of Akt and p‐Akt in CFTR*‐*overexpressing tumours compared to control tumours (Figure 5D). Altogether, these results suggest that CFTR suppresses apoptosis via Akt/Bcl2 pathway.

### 3.5 Expression levels of CFTR are increased in glioblastoma patients

**FIGURE 6:**
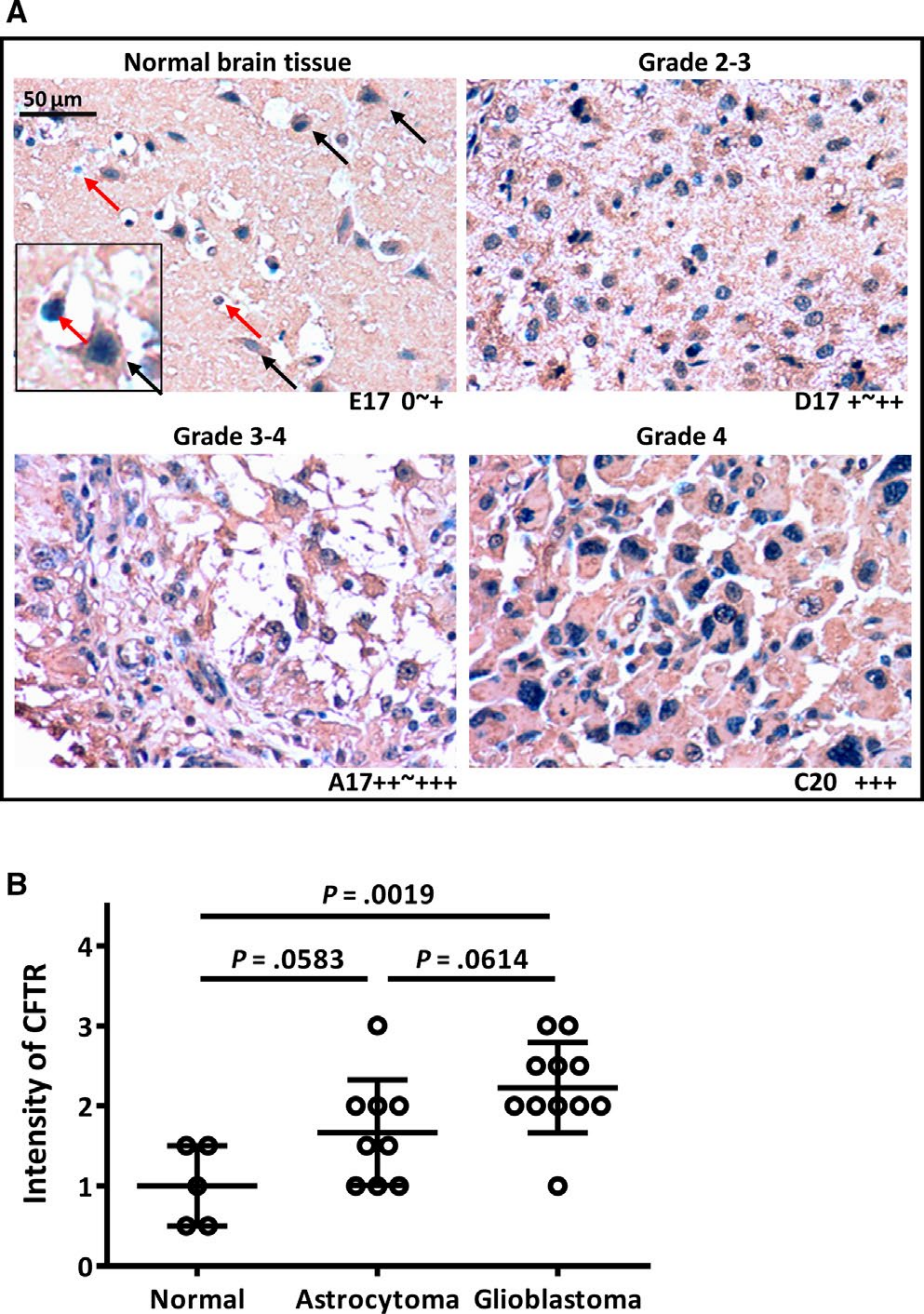
Cystic fibrosis transmembrane conductance regulator (CFTR) is highly expressed in glioblastoma. A, Immunochemical staining of CFTR in samples from tissue microarray (US Biomaxs). The results show that the expression level of CFTR is lower in normal brain (black arrow: neuron; red arrow: astrocyte). In addition, the expression of CFTR shows a relative low to middle intensity in low‐grade (grade 2‐3) astrocytomas comparing to the high‐grade (grade 3‐4 & grade 4). B, Quantification data summarizing the expression level of CFTR in normal and glioma patients

Having established that CFTR regulates apoptotic response and promotes glioma progression, we asked whether the expression level of CFTR is significantly increased in malignant glioma patients and correlates with glioma grades. To answer this question, we examined the protein expression levels of CFTR in a cohort of tissue microarray samples which include 5 cases of normal cerebellum and 20 cases of malignant gliomas using immunochemical staining. The immunochemistry result showed that in normal brain, CFTR was expressed in the neurons at both membrane and cytoplasm (Figure 6A, left panel, Figure S4). The staining was relatively low in astrocytes, which was characterized as cells with naked nuclear and round shape (red arrow compared to black arrow). The expression level of CFTR in malignant glioma is higher than that in normal brain (Figure 6B; Figure S4). In all tested tumours (Table S2, total 20 cases), very weak staining of CFTR was shown in 3/20 of tumours (3/11 in female and 0/9 in male), middle intensity of CFTR was observed in 11/20 of tumors (5/11 in female and 6/9 in male), and high intensity of CFTR was observed in 6/20 of tumors (3/11 in female and 3/9 in male). There was no significant difference in CFTR staining between males and females. Of note, CFTR expression showed a relative low to middle intensity in the low‐grade (grade 2‐3) astrocytomas compared to the high‐grade (grade 3‐4 & 4) astrocytomas or glioblastomas (Figure 6B; Figure S4; Table S2). While we do not find a significant difference in the expression level between normal brain and astrocytoma, the expression levels of CFTR are significantly higher in glioblastoma patients compared to normal brain. These findings suggest that the expression levels of CFTR are significantly increased in glioblastoma.

## 4 DISCUSSION

Emerging evidence suggests that CFTR plays critical roles in modulating a wide variety of cellular processes involved in cancer development, such as cell growth, survival, migration and invasion.^10^, ^11^, ^12^, ^13^, ^15^, ^27^ However, it should be noted that almost all of these studies focused on carcinoma, probably due to the fact that CFTR was thought to be exclusively expressed in epithelial cells. As a membrane protein, CFTR is directly linked to the establishment of cellular polarization, and the establishment and preservation of the structural arrangement of epithelial cells.^28^ On the other hand, polarity proteins have been well‐established as cellular targets of a mounting list of oncogenes and/or tumour suppressors.^29^, ^30^, ^31^ Thus, it is not surprising that CFTR regulates cell growth and cell migration in multiple carcinomas via distinctive signalling pathways.^32^ Despite these findings, the role of CFTR in non‐epithelial cell‐derived cancers is completely unknown. The present study has demonstrated that CFTR is expressed in malignant gliomas. In addition, we have unveiled a tumour promoting role of CFTR in malignant gliomas via up‐regulation of Akt/Bcl2‐mediated anti‐apoptotic pathway.

The correlation of CFTR with PI3K/Akt pathway has been observed in both physiological and pathological conditions in previous studies. Aberrant activity of Akt has been observed in CF cells and mice.^33^, ^34^, ^35^, ^36^ For instance, the macrophages derived from CF mice exhibited a significant decrease of Akt phosphorylation at serine 473 compared with those derived from wild‐type mice after LPS challenge,^34^ which might contribute to the extravagant inflammatory response manifested by CF mice. The same study also demonstrated that reactivation of the Akt pathway in CF macrophages ameliorated hyper‐inflammation in the lung of *CFTR*‐deficient mice. Accordingly, insulin‐induced activation of Akt1 and Akt2 signalling was diminished in human airway epithelia expressing F508del‐*CFTR* compared with the cells expressing wild‐type *CFTR*.^35^ Alternatively, in cancer cells, dysregulation of channel function or knockdown of *CFTR* gene inhibited cell viability and autophagy of LNCaP/CP, a prostate cancer cell line, via activation of Akt/mTOR signalling.^36^ In the current study, we have shown that PI3K/Akt pathway mediates the effect of CFTR on apoptosis regulation in glioma cells. More interestingly, we have observed that the regulatory effect between CFTR and PI3K/Akt pathway is not one directional, as suppression of PI3K/Akt pathway down‐regulates CFTR expression as well (Figure 5B). This finding is in line with the previous study showing that genistein, a promising pharmaceutical drug for CF patients, activates CFTR via the induction of PI3K/Akt‐dependent pathway in duodenal epithelial cells.^37^, ^38^ Thus, it is plausible that CFTR and PI3K/Akt pathway constructs a positive feedback loop to promote glioma development. PI3K/Akt pathway regulates Bcl2 family proteins including both pro‐apoptosis genes and anti‐apoptosis genes, depending on the cellular and environmental context.^39^ In our study, we have found that CFTR up‐regulates Bcl2 more than Bax via PI3K/Akt pathway, thus decreases the ratio of Bax/Bcl2 and makes the glioma cells less susceptible to apoptotic signals. Taken together, based on our findings and previous study, it is plausible that CFTR promotes glioma progression via up‐regulation of Akt/Bcl2‐mediated anti‐apoptosis pathway.

Although accumulated evidence has indicated the involvement of PI3K/Akt pathway in CFTR*‐*mediated signalling and the related physiological and pathological events, how CFTR regulates PI3K/Akt pathway has been elusive. In this study, we show that suppression of channel function itself represses cell viability (Figure 2). Aberrant channel function breaks the ion homeostasis of cells by emitting ions and organic osmolytes into the extracellular space, which creates osmotic driving force for water exit.^40^ Therefore, apoptotic volume decrease (AVD), an essential initial step during apoptosis, is induced.^41^ In cancer cells, it has been demonstrated that suppression of AVD strongly attenuates apoptosis.^42^ CFTR has also been reported as a volume‐regulated Cl^‐^ channel and involved in the regulation of AVD,^43^ thus, it is possible that aberrant Cl^‐^/HCO_3_^‐^ flux due to CFTR dysfunction leads to alteration of cell volume, which further activates Akt pathway in glioma cells. Besides its well‐established channel function, CFTR can regulate various signalling pathways and cell behaviour via other mechanisms. For instance, studies from both other groups and ours have revealed that CFTR suppresses tumour progression via PDZ domain‐dependent protein‐protein interaction.^10^, ^23^, ^44^, ^45^, ^46^ Moreover, multiple signalling pathways and molecules that are involved in the cancer are regulated by CFTR, such as NF‐κB,^23^ β‐catenin^45^ and miRNAs.^11^ Of note, mir‐155‐mediated up‐regulation of PI3K/Akt pathway was reported to be involved in the hyper‐activation of inflammatory response in CF lung epithelial cells.^33^ Subsequent study further revealed that mir‐155 is CFTR‐dependent miRNA, which mediates the up‐regulation of inflammatory cytokine IL‐8.^47^ These results suggest that mir‐155 might mediate the regulatory effect of CFTR on Akt pathway. On the other hand, Sun et al reported that CFTR regulated mitogen‐activated protein kinase (MAPK) pathway via its interaction with adherent junction molecule AF‐6/afadin.^13^ Given that MAPK pathway cross‐talks with Akt pathway in the regulation of cancer development, it is plausible that CFTR modulates Akt/Bcl2 via activation of MAPK. Indeed, MAPK inhibitor can rescue DeltaF508‐CFTR function or expression in epithelial cells,^48^ suggesting a negative regulatory loop between CFTR and MAPK signalling. In our study, we have noticed that CFTR is mainly expressed in cytoplasm, but not at the membrane. Moreover, CFTR activator forskolin (10 μmol/L) cannot rescue the suppressed cell proliferation in CFTR‐knockdown cells (data not shown). On the other hand, it should be noted that CFTRinh172 acts on the cytoplasmic side of the plasma membrane; therefore, one cannot exclude the possibility that besides the channel blocker function, the inhibitor itself disrupts the normal configuration of CFTR and its interaction with other proteins. Based on these observations, we speculate that besides its channel function, other mechanisms are underlying the regulatory effect of CFTR on Akt/Bcl2 pathway.

The PI3K/Akt/mTOR pathway represents an excellent sample of pathway redundancy in biological systems, especially in cancer cells. Indeed, cancer cells are capable of taking advantage of pathway redundancy and routes of feedback to maintain their function and thus escape from drug‐induced cytotoxicity. While PI3K/Akt/mTOR pathway is activated in many types of cancer, little success has been achieved in cancer treatment by targeting the pathway, as tumours eventually evade repression of this pathway. In particular, constitutive PI3K/Akt pathway activation is a hallmark of glioblastomas; however, results from clinical trials by using PI3K/mTOR inhibitors are disappointing.^49^ Therefore, identification of novel targets along this pathway is required for developing alternative therapeutic strategies to suppress this pathway in glioblastoma treatment. We have found in this study that CFTR protein expression is significantly higher in glioblastoma patients compared to low‐grade astrocytoma patients, supporting the promoting role of CFTR in glioma development. In summary, our finding showing that CFTR regulates Akt/Bcl2‐mediated anti‐apoptotic pathway, warrants future investigations into the potential of using CFTR as a therapeutic target and exploring combination therapy to target PI3K/Akt pathway in the treatment of glioblastomas.

## CONFLICT OF INTEREST

The authors declare no conflict of Interest.

## AUTHORS' CONTRIBUTION

Zhao MY and Zhang JT contributed to experimental design and preformation, collection and/or assembly of data, data analysis and interpretation, and manuscript writing. Huang WQ, Dong JD, Guo JH, U KP, Weng Z and Liu S contributed to experimental preformation, collection and/or assembly of data, data analysis and interpretation. Chan HC contributed to provision of study material, financial support and conception of the study. Feng H contributed to provision of study material, data analysis and interpretation, and conception of the study.Jiang X contributed to conception and experimental design, financial support, provision of study material, data analysis and interpretation, and manuscript writing.

## Supporting information

Fig S1‐S4

Table S1‐S2

## Data Availability

The data that support the findings of this study are available from the corresponding author upon reasonable request.
