# Supplementary figures and images for: CFTR promotes malignant glioma development via up‐regulation of Akt/Bcl2‐mediated anti‐apoptosis pathway
Source: J Cell Mol Med. 2020 May 28;24(13):7301–12. doi: 10.1111/jcmm.15300 (PMC7339181; doi:10.1111/jcmm.15300)

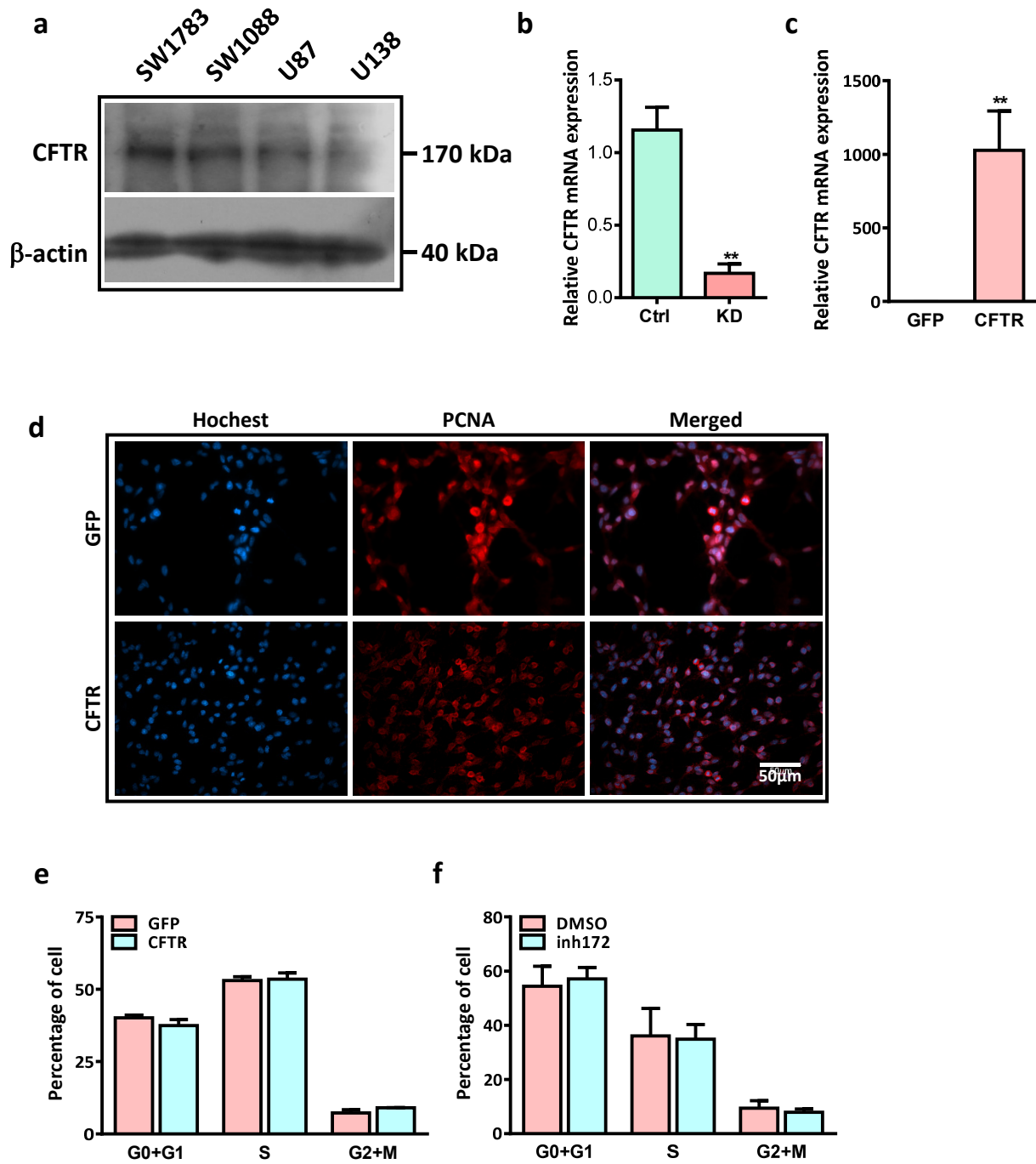

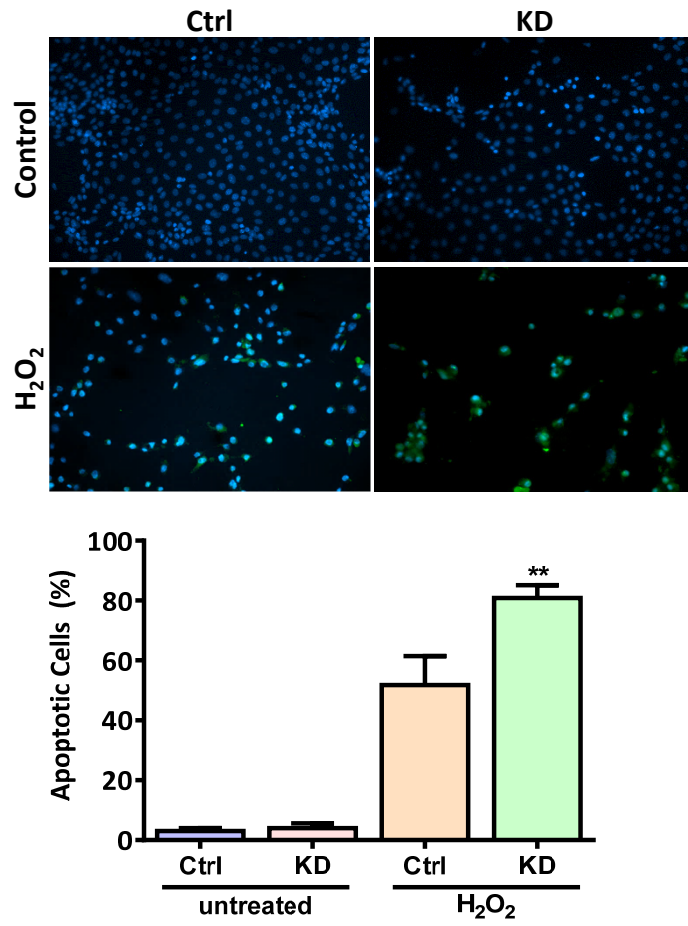

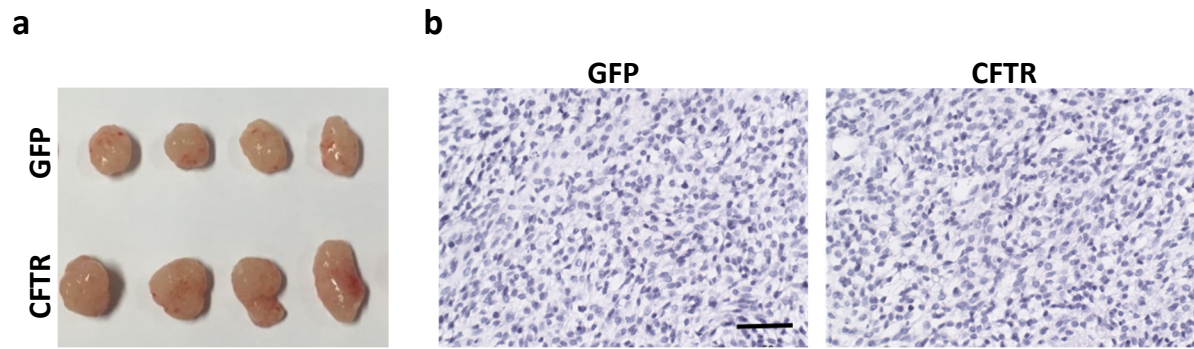

Normal

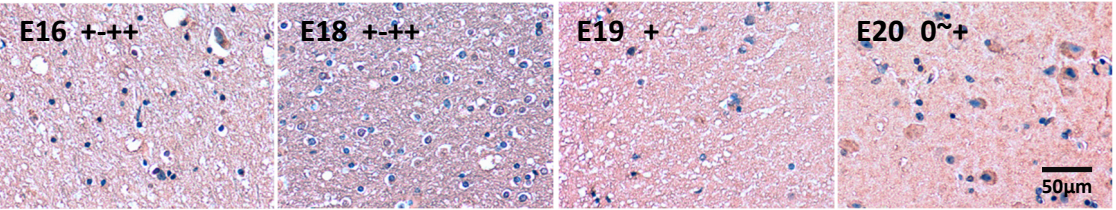

Astrocytoma

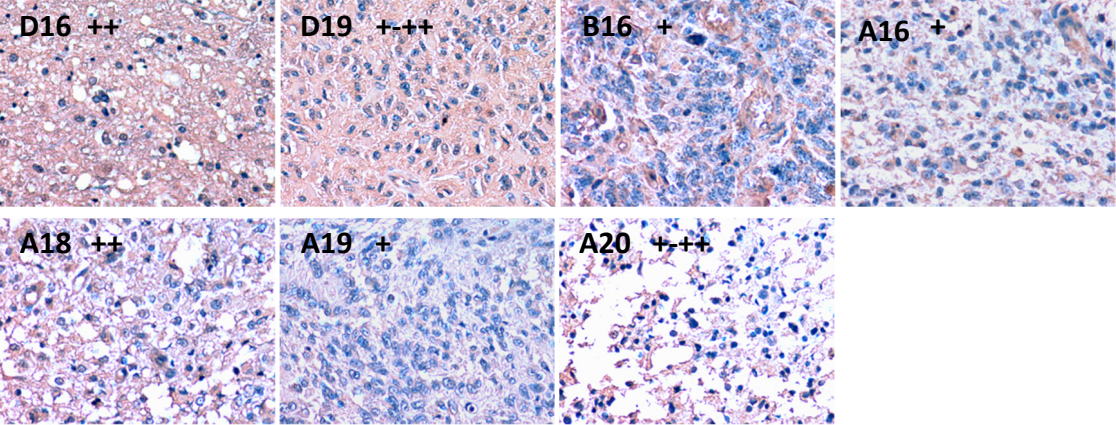

Glioblastoma

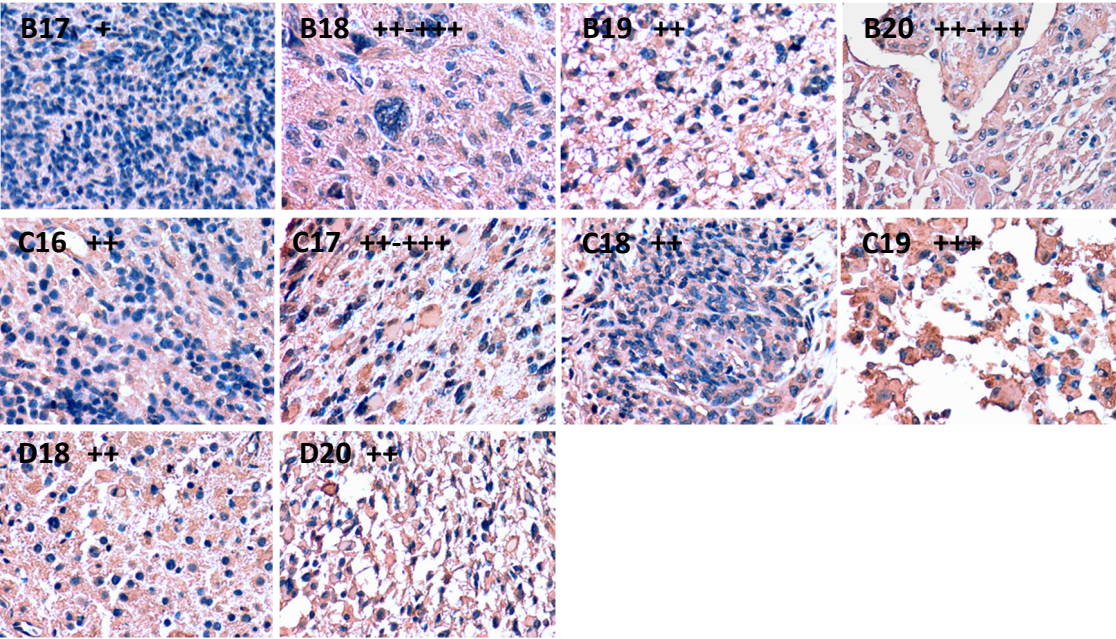

Supplement: Supplementary file 1 — Fig S1‐S4 [file JCMM-24-7301-s001.pdf]
