# Supplementary material for: CFTR promotes malignant glioma development via up‐regulation of Akt/Bcl2‐mediated anti‐apoptosis pathway
Source: J Cell Mol Med. 2020 May 28;24(13):7301–12. doi: 10.1111/jcmm.15300 (PMC7339181; doi:10.1111/jcmm.15300)
Supplement: Supplementary file 2 — Table S1‐S2 [file JCMM-24-7301-s002.pdf]

Supporting Table 1: Primers list used in this study

Gene	Primer sequence (5’-3’)		size
CFTR (For qPCR)	forward	GTGTGATTCCACCTTCTCCAA	149bp
	reverse	GCCTGGCACCATTAAAGAAA	
GAPDH (For qPCR)	forward	AGGGTCATCATCTCTGCC	245bp
	reverse	CCATCACGCCACAGTTTC	
CFTR (452bp)	forward	AAAACTTGGATCCCTATGAAC	452bp
	reverse	GTGGGGAAAGAGCTTCAC	
CFTR (248bp)	forward	CCATTTTTGGCCTTCATCAC	248bp
	reverse	GGCAGACGCCTGTAACAACT	
Bcl2	forward	ATTTCCTGCATCTCATGCCAAGGG	90bp
	reverse	TGTGCTTTGCATTCTTGGACGAGG	
p53	forward	GGATGATTTGATGCTGTC	400bp
	reverse	CACAACCTCCGTCATGTG	
Bax	forward	ATGGACGGGTCCGGGGAGCAG	380bp
	reverse	TCAGCCCATCTTCTTCCAGAT	
GAPDH	forward	TCCCATCACCATCTTCCAG	515bp
	reverse	TCCACCACTGACACGTTG	
ß-actin	forward	GTGGGGCGCCCCAGGCACCA	540bp
	reverse	CTCCTTAATGTCACGCACGATTTC	

Supporting Table 2: Information of patients.

No.	SEX	AGE	ORGAN	PATHOLOGY	GRADE	TYPE	IHC INTENSITY
A16	F	50	Brain	Astrocytoma	3-4	Malignant	+
A19	F	46	Brain	Astrocytoma	3-4	Malignant	+
B16	F	61	Cerebrum	Astro-glioblastoma	3-4	Malignant	+
B17	F	31	Cerebrum	Glioblastoma	4	Malignant	+
B20	F	41	Cerebrum	Glioblastoma	4	Malignant	++~+++
C16	F	55	Cerebrum	Glioblastoma	4	Malignant	++
C17	F	32	Cerebrum	Glioblastoma	4	Malignant	++~+++
C19	F	59	Cerebrum	Glioblastoma	4	Malignant	+++
D16	F	41	Cerebrum	Astrocytoma	2	Malignant	++
D17	F	50	Cerebrum	Astrocytoma	3	Malignant	++
D19	F	49	Cerebrum	Astrocytoma	3-4	Malignant	+~++
A17	M	51	Brain	Astrocytoma	3-4	Malignant	+++
A18	M	56	Brain	Astrocytoma	3-4	Malignant	++
A20	M	35	Brain	Astrocytoma	3-4	Malignant	+~++
B18	M	40	Cerebrum	Glioblastoma	4	Malignant	++~+++
B19	M	55	Cerebrum	Glioblastoma	4	Malignant	++
C18	M	39	Cerebrum	Glioblastoma	4	Malignant	++
C20	M	44	Cerebrum	Glioblastoma	4	Malignant	+++
D18	M	53	Cerebrum	Glioblastoma	4	Malignant	++
D20	M	48	Cerebrum	Glioblastoma	4	Malignant	++
E17	M	26	Brain	Normal brain tissue	-	Normal	0~+
E16	F	28	Brain	Normal brain tissue	-	Normal	+~++
E18	F	42	Brain	Normal brain tissue	-	Normal	+~++
E19	F	38	Brain	Normal brain tissue	-	Normal	+
E20	F	50	Brain	Normal brain tissue	-	Normal	0~+
